# Training in Compensatory Strategies Enhances Rapport in Interactions Involving People with Möbius Syndrome

**DOI:** 10.3389/fneur.2015.00213

**Published:** 2015-10-08

**Authors:** John Michael, Kathleen Bogart, Kristian Tylén, Joel Krueger, Morten Bech, John Rosendahl Østergaard, Riccardo Fusaroli

**Affiliations:** ^1^Department of Cognitive Science, Central European University, Budapest, Hungary; ^2^Center for Subjectivity Research, Copenhagen University, Copenhagen, Denmark; ^3^Interacting Minds Centre, Aarhus University, Aarhus, Denmark; ^4^School of Psychological Science, Oregon State University, Corvallis, OR, USA; ^5^Center for Semiotics, Aarhus University, Aarhus, Denmark; ^6^Department of Sociology, Philosophy, and Anthropology, University of Exeter, Exeter, UK; ^7^Department of Pediatrics, Aarhus University Hospital, Skejby, Denmark

**Keywords:** Möbius syndrome, interaction, gesture, rapport, alignment

## Abstract

In the exploratory study reported here, we tested the efficacy of an intervention designed to train teenagers with Möbius syndrome (MS) to increase the use of alternative communication strategies (e.g., gestures) to compensate for their lack of facial expressivity. Specifically, we expected the intervention to increase the level of rapport experienced in social interactions by our participants. In addition, we aimed to identify the mechanisms responsible for any such increase in rapport. In the study, five teenagers with MS interacted with three naïve participants without MS before the intervention, and with three different naïve participants without MS after the intervention. Rapport was assessed by self-report and by behavioral coders who rated videos of the interactions. Individual non-verbal behavior was assessed via behavioral coders, whereas verbal behavior was automatically extracted from the sound files. Alignment was assessed using cross recurrence quantification analysis and mixed-effects models. The results showed that observer-coded rapport was greater after the intervention, whereas self-reported rapport did not change significantly. Observer-coded gesture and expressivity increased in participants with and without MS, whereas overall linguistic alignment decreased. Fidgeting and repetitiveness of verbal behavior also decreased in both groups. In sum, the intervention may impact non-verbal and verbal behavior in participants with and without MS, increasing rapport as well as overall gesturing, while decreasing alignment.

## Introduction

Möbius syndrome (MS) is a form of congenital facial paralysis – typically complete and bilateral – resulting from maldevelopment of the sixth and seventh cranial nerves (1). MS is extremely rare, occurring in 2–20 births/million (2). Given the centrality of the face for expressing emotions and other mental states, and for communicating other non-verbal social cues, it is natural to expect that people with MS may experience difficulties in their social interactions.

Indeed, some studies corroborate these expectations. One study by Briegel (3) found that people with MS had a significantly higher incidence of inhibition, introversion, and interpersonal sensitivity (feelings of inadequacy and inferiority), a lower satisfaction with life, a less pronounced achievement orientation, and a greater tendency toward psychological distress. However, in a larger study, Bogart and Matsumoto (4) compared 37 Americans with MS to 37 matched controls without facial paralysis and found no differences between the groups in depression or anxiety, although they did find that participants with MS had lower social competence. The picture is, thus, far from clear, and some of the differences in results may plausibly be attributed to differences in sample size, measures, or cultural differences in disability stigma (5). The conclusion to be drawn from these studies is that some people with MS manage well, whereas others may benefit from psychosocial support.

In order to identify ways of improving the level of comfort and satisfaction in social interactions of people with MS, it is important to distinguish several (mutually consistent and related) reasons why facial paralysis may lead to difficulties in social interaction, ranging from impairment and processing of facial expression, to the way this resonates in the interaction and impacts the interlocutor. First of all, some researchers have speculated that facial mimicry may contribute to social understanding by playing a central role in the identification of others’ emotions on the basis of their facial expressions (6, 7). The suggestion is that in order to identify what type of emotion an individual is experiencing, it may be necessary to facially mimic, or simulate, their facial expression. Thus, individuals with deficits in producing, experiencing, or expressing an emotion may also have a deficit in the face-based recognition of that same emotion when they see it in others [e.g., Ref. (6, 8, 9)]. Given the lack of facial expressivity on the part of people with MS, the simulation model predicts that they may be impaired at face-based emotion recognition – which could explain some of the difficulties they experience with social interaction.

There has been some research bearing upon this hypothesis, so far with mixed results. Most recently, Bate et al. (10) reported that five of six participants with MS were impaired in at least one of the three tasks they employed to assess face-based emotion recognition. However, only one of the participants in this study was impaired on a task that involved imagining facial expressions of emotions and answering questions about them. As the authors point out, this is not consistent with the simulation model. Moreover, in another recent study, Bogart and Matsumoto (11) compared 37 Americans with MS to 37 matched American controls without facial paralysis, and found no significant difference in performance on a face-based emotion recognition task. The differences between these findings may be due in part to differences in the methodologies employed in the two studies. It is possible, for example, that people with MS employ alternative means for face-based emotion recognition to compensate for the absence of facial mimicry. If so, one might expect them to perform equally well if not better than controls on measures, such as detailed facial processing, but worse on others, for example, facial processing under time pressure.

In any case, several other factors are likely to be equally or even more important than any deficit in face-based emotion recognition on the part of people with MS. First, it is highly probable that *other* people have difficulties in dealing with their interlocutors’ facial paralysis, creating awkward interactions. Since people are accustomed to receiving information about others’ mental states from their facial expressions, the absence of this expected information may interrupt an interaction partners’ facial mimicry and cause him or her to feel uncomfortable or confused about what the person with MS is thinking or feeling. This conjecture is corroborated by evidence that individuals with facial movement disorders, such as MS or Parkinson’s disease, are often perceived as unhappy, unfriendly, depressed, disinterested, or unintelligent (12–15), making others less likely to pursue engagement and friendships with them (16). In sum, the social difficulties experienced by many people with MS may lie partially with their interaction partners without MS, who may for various reasons feel uncomfortable or confused. Consequently, the smooth flow of interaction is interrupted through their uncertainty about how to interact in what is for them a new and sensitive situation. In turn, children with MS might find themselves in more awkward and difficult interactions than non-MS children, potentially impacting the development of their social skills (9, 10, 17)^1^, and perhaps also leading to an impoverishment of their own emotional experience (19).

In examining social interactions, it would, thus, be just as naïve to focus narrowly on the interaction partner with MS as it would be to focus narrowly on the interaction partner without MS, as their individual behaviors are not generated in a vacuum, but resonate with and constrain each other (20, 21). In other words, some of the most relevant consequences of MS for social interaction may stem from the effects that the lack of facial expressivity has upon the dynamics of the interaction itself. After all, people with MS are deprived of an important means for displaying emotional appraisal, giving feedback and indicating understanding. Facial expressivity is, thus, an important component in the continuous reciprocal adaptations between interlocutors that supports the alignment and sharing of emotional states, perspectives and attitudes, and facilitates understanding. Although there is a broad consensus about the importance of reciprocal facial expressivity for social interaction and social cognition, there are also many open questions. To what extent are facial expressions flexibly coordinated, deliberately controlled and potentially replaced, and how do they interact with other aspects of interactive behavior, such as vocal prosody and hand gestures (22–24)? This latter cluster of questions may be especially relevant for people with MS, who may benefit by compensating for their limited facial expressivity with some other form of expressivity.

### Use of compensatory strategies

Although many people with MS report difficulties in their social interactions, others clearly are professionally and personally happy, and have little trouble with social interactions. Bogart et al. (25) suggest that, as observed in many neuropsychiatric conditions (26, 27), some people with MS may have developed compensatory strategies to manage their social interactions. To understand how they achieve this would be valuable, as it would potentially help other people with MS (as well as people with facial paralysis and facial difference arising from other causes) in adopting the compensatory strategies employed by these more socially successful individuals.

Anecdotal evidence suggests that such compensatory strategies likely include expressing oneself more with hand gestures and body language as well as prosody (19). Moreover, in qualitative interviews, many people with MS reported that they deliberately compensated by using eye contact to display confidence, and prosody, body language, and verbal disclosure to express emotion (25, 28, 29). In a behavioral study, Bogart et al. (30) found that people with MS used more of these compensatory expressive behaviors than people with acquired facial paralysis, who may not be as well adapted to their impairment due to the later stage at which they acquired it. Moreover, there is evidence that social skills training programs can benefit people with facial differences who experience problems with social interaction (31). Thus, developing a social skills workshop that promotes the use of alternative channels, such as prosody, gestures, and verbal disclosure, to communicate emotion may benefit people with MS, or other types of facial paralysis, or facial difference.

The conjecture that bodily and prosodic expressivity can substitute for facial expressivity is also supported by the results of a recent study in which Bogart et al. (13) asked participants to judge the emotions of people with facial paralysis based on all communication channels or a limited number of channels (e.g., face only or voice + speech + body, etc.). Results showed that participants were better able to assess the emotions when more communication channels were available, suggesting that they were perceiving in a holistic manner, taking into account information from multiple channels. Furthermore, they formed more positive impressions of individuals who used more compensatory means of expression.

### Interactive alignment and rapport

In addition to communicating and sharing emotions, an increase in bodily expressivity may support social interaction via *interactive alignment*, which can be defined as the tendency of interaction partners spontaneously to imitate each other’s patterns of movement and speech (32). Interactive alignment has been observed in many aspects of interactive behavior from the spontaneous temporal synchronization of movements (33), to expressive and postural mimicry (34, 35), and to multiple dimensions of linguistic coordination (32, 36–38) and has been speculated to subserve important social functions. Alignment has been found to promote rapport, defined as a state of connectedness involving mutual attentiveness, positivity, and responsiveness (39–43), and thereby also increase participants’ willingness to cooperate with each other (44, 45). Thus, a possible lack of alignment in interactions involving people with MS – due to the impaired facial expressivity in one interlocutor and/or to general uneasiness – could have significant consequences, leading to lower rapport and coordination with potentially impaired reciprocal understanding.

However, a handful of studies point to a different set of predictions. Borrie and Liss (46) have shown that individuals tend to align their verbal behavior more if one of the interlocutors has a speech impairment. Analogously, in an analysis of psychotherapeutic sessions, Reich and colleagues highlighted the presence of higher prosodic alignment for poorer relationships between therapist and client as well as for greater client distress (47). These findings imply that alignment might decrease after an intervention that successfully reduced the level of social anxiety and the need to compensate for a lack of interactional fluency. In a similar vein, it has been argued that in many forms of interactions, complementary patterns and not just alignment of behavior might be a better predictor of rapport and efficacy of the interaction. In good interactions, the prosodic pattern indicating a question will be followed by the prosodic pattern indicating an answer; an impromptu rising of the voice could be followed by a lowering of the intensity to avoid an emotional escalation; and interlocutors will not repeat each other’s words, but contribute new topics to the conversations (20, 21, 24, 38).

In other words, a change in interactive alignment might be crucial in assessing the efficacy of the interaction, but it is unclear whether increased or decreased alignment would be the desired development.

### The current study

As the foregoing remarks indicate, there are at least three factors that may present challenges to people with MS in social interactions: (1) emotion perception and recognition may be difficult for some people with MS; (2) the absence of facial expressivity can make it difficult for *others* to understand *them*, and can create a cold or disinterested impression; and (3) their facial impairment may interfere with one central medium for interactive alignment processes which can engender rapport.

Insofar, as these three factors may present challenges to people with MS, they also provide three entry points for a social skills intervention designed to help people with MS. We, therefore, incorporated exercises targeting all three factors into an intervention for teenagers with MS. The main aim of the present study was to test the efficacy of this workshop. Specifically, we expected that the level of rapport experienced in social interactions would increase from before the intervention to afterward, as measured by independent coders and by self-report. A further aim of the study was to identify the mechanisms responsible for any such increase in rapport – focusing not only on the behavior of the participants with MS but also on their interaction partners without MS and on the dynamics of the interaction itself. We predicted that all participants would show greater expressivity of their body and voice after the intervention than before, as measured by behavioral coders rating the videos, and also that the level of expressivity would correlate with rapport. Moreover, we hypothesized that interactive alignment would change after the intervention, and that it, too, would correlate with rapport. Either direction of change would be supported by the literature. Consequently, we endeavored to use our findings to further articulate our understanding of interactive alignment.

## Materials and Methods

### Design overview

The present study was built around an intervention designed to train people with MS to adopt alternative strategies to compensate for the unavailability of facial expression in social interactions and to increase social comfort. The experiment was conducted as a 2-day workshop at the Pindstrup Centre in Denmark, and was approved by the local chapter of the Danish Research Ethics Committee. In order to assess possible changes in expressive behavior and rapport following the intervention, participants completed dyadic interactions with naïve interlocutors both at baseline and after the intervention.

### Participants

We recruited five teenagers (three females) with MS between 14 and 19 years old (mean age 16.20, SD = 1.92) (henceforth “MS participants”). We judged that this age group would stand to benefit most from the social skills intervention, as people with MS report their teenage years being the most challenging (25, 28). MS participants were observed to have moderate to severe facial paralysis. They were referred for the study by a physician (the sixth author of the study, who is a trained neurologist and pediatrician) who affirmed that they were cognitively able, and all were attending mainstream school at a level appropriate to their age. Using a university database as well as word of mouth, we also recruited 10 people without MS (five females) in the same age range (mean age 17.70, SD = 2.79). All 10 non-MS participants were informed that they would be asked to engage in brief, videotaped interactions with teenagers with bilateral facial paralysis. All participants (and, for those under 18, their parents) gave their informed written consent. Of the 10 participants without MS (henceforth “non-MS participants”), five participated on the first day (before the intervention) and five on the second day (after the intervention). The reason for this was that if the non-MS participants had participated on both days, a confound would have arisen insofar as our results might have been explained by the increasing familiarity with the procedure, with the participants with MS, and with MS in general, rather than by the intervention. Thus, each MS participant interacted with six different non-MS participants, and each non-MS participant interacted with three MS participants. Multiple interactions with different interlocutors were important to enable the individual variability of MS and non-MS participants to be accounted for in the statistical analysis.

### Procedure

#### Intervention

The intervention was based on the social skills workshop developed by the UK organization for people with visible differences, *Changing Faces*. A randomized controlled trial found that burned adolescents who received the intervention were significantly less withdrawn, had fewer somatic complaints, and less behavioral problems compared to the control group at the 1-year follow-up (31). To address the unique expressive challenges of facial paralysis, the intervention was modified to encourage the use of compensatory expressive behaviors. The intervention was designed by the second author and delivered by the second and fifth authors of the study, both psychologists. The 2-day group workshop involved instruction (targeting non-verbal and verbal expression and recognition, conversational skills), group discussion of personal experiences, role-playing, group activities, and writing exercises. The first day involved a group discussion of different modes of expression, including the face, body, prosody, gesture, posture, backchannel responses, and style. They were instructed that people typically look to the face as the primary source of information, but that with MS, other modes of communication can be used instead. The role of mimicry in communication was also discussed. Group activities involved observing and recognizing emotions in themselves and others. For example, one exercise was a charades game in which participants had to express and recognize emotions without naming them. At the end of the first day, participants were assigned homework to interact with one new person and with a family member, and to observe their communication skills with each person. On the second day, they discussed their observations from their homework assignment. Then, participants discussed their feelings during social interactions and how others might feel when meeting them. Communication skills to put others at ease, including behaving in a friendly, confident manner, were trained. In a writing assignment, participants prepared several ways to quickly explain MS to others and shared them with the group. Additionally, participants generated personal examples of social situations that were successful and that were challenging. They then role-played the challenging situations and discussed what could have been done differently. The workshop manual is available upon request.

#### Dyadic Interactions

During test-phase interactions, the participants with MS were paired with non-MS interlocutors. Pairing was randomized and blocked to ensure that participants of similar ages were paired (the maximum age difference was 2 years). In order to elicit emotional communication in a naturalistic way, the interlocutors were instructed to tell each other about recent, enjoyable experiences. Each MS participant went through six interactions in total, three before the intervention and three afterward. Each interaction lasted approximately 6–8 min and was considered as a whole. Participants sat in armless chairs to allow for unrestricted arm movement. There were two video cameras per dyad; one to record each participant.

#### Measures

In order to assess the impact of intervention on the quality of the interactions, we employed multiple measures of rapport as well as verbal and non-verbal behaviors in all interactions before and after the intervention. Based on previous research involving behavioral coding of compensatory expression of people with facial paralysis, fidgeting, and head and face movements were expected to be particularly relevant non-verbal behaviors (30). Pitch and speech rate were chosen amongst the most commonly reported measures of prosodic coordination in conversations, and for their reported correlation with rapport and efficacy of interpersonal coordination (21, 47–49). We also administered questionnaires to assess the social competence (50) and social anxiety (51) of MS participants at three points: immediately before the experiment, immediately afterward, and 6 months later.

### Rapport

All participants completed self-report rapport scales (52) after each interaction. An example item is “did you feel in rapport with him or her?” Responses to items were averaged, with scores ranging from 1 to 6, with 6 indicating higher rapport. To measure observed rapport, the two video recordings from each interaction were synced and spliced together using Adobe Premiere Pro CS6 with a handclap as a sync point. Thus, each interlocutor could be viewed at the same time in a split screen. Based on the method of Bernieri (41), five behavioral coders who were blind to the hypotheses viewed these videos with the sound turned off and rated the shared liking of the dyad on a scale from 1 to 5. Inter-rater reliability (Chronbach’s α) was 0.70.

### Non-verbal behaviors

Five behavioral coders blind to the hypotheses watched the videos and scored the quality, intensity, and frequency of gestures and bodily cues used by both interaction partners. The coding system employed in Bogart et al. (30) was used to measure the expressivity of individuals with facial paralysis and of their interaction partners. Coders rated the following four items: gesture (α = 0.86), fidgeting (α = 0.78), head movements (α = 0.68), and facial expressivity (α = 0.93). Ratings were performed on a 1–5 scale, with higher numbers indicating more expressivity.

### Verbal behaviors

Pitch and speech rate were extracted from the audio recordings of the conversations employing a semi-automated process. First, a research assistant blind to the hypotheses carefully watched the videos and manually tagged onsets and offsets of the interlocutors’ speech turns to ensure accurate speaker attribution. The manual tags were then adjusted to a 10-ms precision scale through an automated analysis of pitch presence/absence and intensity changes using Matlab (Mathworks Inc.). We extracted pitch (fundamental frequency, Hertz) within normal human voice range (70–400 Hz) and intensity (decibel) at a 10 ms scale using Praat (53) and corrected for artifactual octave jumps. We then calculated speech rate as estimated syllables per minute. To do so, we first isolated all utterances by interlocutor, excluding speech overlapping, and individuated voiced peaks in intensity as proxies for vowel onsets according to the procedure in de Jong and Wempe (54). In order to be able to capture shared dynamics between the two interlocutors, we needed uniformly sampled time-series. The vowel onsets were, therefore, converted to estimated syllables per minute sampled every 333 ms, following the validated procedure in Wallot et al. (55).

### Analysis

#### Impact of Intervention on Rapport

We chose two measures of rapport: the mean of self-reported rapport from the two interlocutors, and rapport as assessed by the coders. First, we measured the relation between self-reported rapport in interlocutors using a mixed effect correlation with identity of the individual interlocutors as random factor, and we tested the impact of intervention by comparing the base model with a model including a before/after intervention fixed factor (56). We then measured the impact of the intervention on rapport by employing a mixed-effects multivariate model with random intercepts with before/after intervention as fixed factor and the identity of the individual interlocutors as random factors (random intercepts only, as the models would generally not converge with random slopes). Multiple testing was corrected using Student–Newman–Keuls corrections. We report estimates of effect size of the overall model (*R*^2^) and of the fixed factors (*R*^2^ marginal), following the procedure described in Nakagawa and Schielzeth (57). Estimates and confidence intervals of the descriptive statistics were calculated via bootstrapping 1000 times, stratifying by interlocutor identity. The mixed-effects models were calculated using the lme4 package for R (58).

#### Impact of Intervention on Individual Behaviors

First we assessed the impact of intervention on individual behaviors: gesture, fidgeting, facial expressivity, head movement, pitch SD, speech rate mean and SD. Pitch mean was not employed, as it would vary greatly according to the gender and age of the participants. We employed 2-by-2 repeated measures models with two fixed factors (MS vs. non-MS and before vs. after intervention). Interlocutors’ identity was defined as a random factor (random intercepts only, as the models would generally not converge with random slopes). Estimates and confidence intervals of the descriptive statistics were calculated via bootstrapping 1000 times, stratifying by interlocutor identity. Again the mixed effect models were calculated using the lme4 package for R. Descriptive statistics of individual behaviors before and after intervention divided by group are available in Table 1.

**Table 1:** **Descriptive statistics of individual behaviors before and after intervention by group**.

Feature	MS pre-intervention	MS post-intervention	NMS pre-intervention	NMS post-intervention
Gesture (*n* = 15 interlocutors)	1.93 (CI: 1.71, 2.14)	2.55 (CI: 2.38, 2.70)	1.72 (CI: 1.59, 1.85)	2.32 (CI: 2.17, 2.49)
Fidget (*n* = 15 interlocutors)	2.79 (CI: 2.62, 2.94)	2.69 (CI: 2.54, 2.81)	2.31 (CI: 2.17, 2.44)	1.91 (CI: 1.78, 2.06)
Facial expressivity (*n* = 15 interlocutors)	1.54 (CI: 1.48, 1.60)	1.65 (CI: 1.61, 1.70)	3.09 (CI: 2.97, 3.21)	3.40 (CI: 3.20, 3.56)
Head movement (*n* = 15 interlocutors)	2.36 (CI: 2.19, 2.54)	2.59 (CI: 2.49, 2.67)	2.56 (CI: 2.47, 2.65)	2.65 (CI: 2.50, 2.79)
Pitch SD (*n* = 15 interlocutors)	42.07 (CI: 36.68, 47.72)	40.95 (CI: 35.59, 46.41)	45.16 (CI: 39.61, 51.32)	40.16 (CI: 35.50, 45.42)
Speech rate mean (*n* = 15 interlocutors)	151.65 (CI: 130.81, 173.82)	141.96 (CI: 104.41, 167.76)	144.82 (CI: 119.81, 158.83)	150.68 (CI: 123.87, 171.11)
Speech rate SD (*n* = 15 interlocutors)	118.74 (CI: 107.42, 133.22)	112.66 (CI: 88.38, 145.20)	122.57 (CI: 117.47, 132.56)	119.87 (CI: 105.63, 123.88)

#### Impact of Intervention on Alignment

In order to assess the presence of coordination of non-verbal behaviors between interlocutors, we employed mixed effect models evaluating the relation between the coded levels (e.g., gesture) in the two interlocutors, using interlocutor identity as a random effect. This enabled us to assess whether the actual interaction created statistically significant alignment between interlocutors. Second, we assessed whether intervention changed the strength of the matching. To achieve this, we examined whether the addition of before/after intervention as an additional fixed factor in the model would statistically improve it and influence the direction of the impact. The more fine-grained nature of our linguistic data enabled us to assess not only the presence of behavioral matching but also whether it was due to the actual dynamics of interaction as opposed to the general structure of conversation. We, therefore, employed cross recurrence quantification analysis (CRQA) (59), a non-linear and more flexible analog of cross correlation, which quantifies shared dynamics between time-series and has been shown to be more sensitive to the dynamics of interpersonal coordination [Ref. (60); for a comprehensive review of the application of CRQA to interpersonal coordination, cf. Ref. (20, 38)]. Relying on the time-series produced by each interlocutor (e.g., a sequence of estimated speech rate regularly sampled over time), CRQA reconstructs the phase space of possible combinations of states and quantifies the structure of recurrence, that is, of the number of instances in which the two time-series are displaying similar dynamics and the characteristics of these instances. This generates several indexes of fundamental frequency and speech rate coordination:

• Level of coordination: defined as the percentage of values that recur (are present) in both time-series independently of the lag [recurrence rate (RR)]
• Stability of coordination, articulated in tendency of recurrence to happen in sequences (as opposed to isolated points being repeated, DET), average length of sequences repeated across time-series (L), and length of longest repeated sequence (LMAX)
• Complexity of coordination: defined as low if all repeated sequences are of the same length, high is repeated sequences vary in length [entropy (ENTR)], thus suggesting that coordination is flexible and not mechanical imitation.

We then tested whether the intervention impacted the level of coordination employing mixed-effects models as in the previous analysis. CRQA was performed using the CRP toolbox for Matlab. Estimates and confidence intervals of the descriptive statistics were calculated by bootstrapping 1000 times, stratifying by interlocutor identity. The mixed-effects models were calculated using the lme4 package for R.

#### Relation between Rapport and Interactional Behaviors

Finally, we assessed the relation between interactional behaviors and rapport. In particular, we attempted to statistically predict self-reported and observer-coded rapport as a function of our measures of verbal and non-verbal interactional behaviors, including measures of alignment. This enabled us to assess (i) how well the behavioral indexes could predict experience of the interaction, (ii) whether employing multiple behavioral indexes adds to our ability to assess the interaction or they all express the same underlying dynamic, and (iii) whether our results would be sensitive to individual baselines, or generalize across participants. To obviate the issues due to the large number of features, for each measure of rapport we used a fivefold cross-validated process (61) in which we first selected a limited amount of minimally overlapping features using ElasticNet (62, 63) and then ran a multiple regression, calculating the accuracy of the model using adjusted R square (Adj *R*^2^) (63). The cross-validation process ensured that the accuracy of the model was calculated only for test datasets on which the model had not been trained. Cross-validation was performed at the level of the interlocutors, meaning that the model was never tested on conversations including interlocutors on which the model was trained. These analyses were performed using the statistics and bioinformatics toolboxes for Matlab.

## Results

### Impact of intervention on rapport

Self-reported rapport was positively correlated between interlocutors: *R*^2^ = 0.42 (CI: 0.26, 0.57), with fixed factor accounting for a marginal *R*^2^ of 0.17 (CI: 0.03, 0.41), *p* = 0.001. The correlation did not statistically change due to intervention (*p* = 0.61). Self-reported rapport did not statistically change after intervention, while the coder-assessed rapport showed a significant increase (cf. Table 2).

**Table 2:** **Impact of intervention on rapport**.

Rapport	Before intervention	After intervention	Difference	*R*^2^	*R*^2^ marginal	*P*-value
Coder-assessed (*n* = 15 interactions)	2.8 (CI: 2.6, 3)	3.3 (CI: 3.1, 3.5)	0.46 (CI: 0.15, 0.77)	0.22	0.15	0.001
Self-reported						
Mean (*n* = 30 interlocutors)	4.5 (CI: 4.4, 4.7)	4.4 (CI: 4.3, 4.7)	−0.1 (CI: −0.2, 0.1)	0.04	≃0	≃1
MS (*n* = 15 interactions)	4.3 (CI: 4, 4.6)	4.4 (CI: 4.1, 4.7)	0 (CI: −0.1, 0.1)	0.03	≃0	≃1
Non-MS (*n* = 15 interactions)	4.8 (CI: 4.7, 4.9)	4.4 (CI: 4, 4.8)	−0.3 (CI: −0.6, 0.1)	0.01	≃0	≃1

### Analysis of individual behaviors

We observed significant main effects for both MS vs. non-MS and before vs. after intervention on individual behaviors (for full descriptive statistics, cf. Table 1). Non-MS participants exhibited higher degrees of gesturing, and facial expressivity as well as fidgeting (but not head movements). They also exhibited a less variable speech rate (cf. Table 3).

**Table 3:** **Main effect of MS participants vs. non-MS participants on individual behaviors**.

Feature	MS	Non-MS	Difference	*R*^2^	*R*^2^ marginal	*P*-value
Gesture (*n* = 15 interlocutors)	1.72 (CI: 1.63, 1.77)	1.93 (CI: 1.78, 2.08)	0.21 (CI: 0.03, 0.38)	0.26	0.14	0.013
Fidget (*n* = 15 interlocutors)	2.31 (CI: 2.20, 2.41)	2.70 (CI: 2.66, 2.90)	0.29 (CI: 0.08, 0.66)	0.08	0.06	<0.001
Facial expressivity (*n* = 15 interlocutors)	1.56 (CI: 1.36, 1.75)	3.10 (CI: 2.78, 3.42)	1.54 (CI: 1.17, 1.92)	0.90	0.07	0.04
Head movement (*n* = 15 interlocutors)	2.42 (CI: 2.20, 2.62)	2.55 (CI: 2.27, 2.84)	0.13 (CI: 0.17, 0.44)	0.06	≃0	0.42
Pitch SD (*n* = 15 interlocutors)	67.78 (CI: 56.80, 77.21)	69.01 (CI: 60.19, 78.15)	1.53 (CI: −3.39, 5.61)	0.35	≃0	0.23
Speech rate mean (*n* = 15 interlocutors)	147.10 (CI: 125.79, 163.88)	150.02 (CI: 132.25, 161.51)	2.81 (CI: −9.02, 13.10)	0.11	≃0	0.27
Speech rate SD (*n* = 15 interlocutors)	114.78 (CI: 103.43, 120.57)	120.86 (CI: 114.62, 124.66)	6.40 (CI: −1.73, 13.34)	0.08	0.06	0.024

After intervention, we observe a statistical increase of gesturing, facial expressivity, and head movements in both groups of interlocutors. Speech rate slowed down, but it exhibited higher variability (cf. Table 4).

**Table 4:** **Main effect of before vs. after intervention on individual behaviors**.

Feature	Before intervention	After intervention	Difference	*R*^2^	*R*^2^ marginal	*P*-value
Gesture (*n* = 15 interactions)	1.82 (CI: 1.65, 2.01)	2.44 (CI: 2.21, 2.62)	−0.57 (CI: −0.85, −0.26)	0.33	0.15	<0.001
Fidget (*n* = 15 interactions)	2.54 (CI: 2.36, 2.74)	2.30 (CI: 2.09, 2.54)	0.30 (CI: 0.02, 0.59)	0.17	0.12	0.005
Facial expressivity (*n* = 15 interactions)	2.31 (CI: 2.05, 2.62)	2.53 (CI: 2.21, 2.81)	−0.27 (CI: −0.58, 0.03)	0.21	0.14	<0.001
Head movement (*n* = 15 interactions)	2.36 (CI: 2.21, 2.56)	2.61 (CI: 2.46, 2.76)	−0.25 (CI: −0.46, 0.03)	0.20	0.11	0.01
Pitch SD (*n* = 15 interactions)	40.54 (CI: 37.02, 43.87)	43.80 (CI: 40.05, 47.73)	−3.26 (CI: −9.61, 3.12)	0.07	≃0	0.16
Speech rate mean (*n* = 15 interactions)	150.43 (CI: 134.45, 162.83)	137.19 (CI: 114.36, 155.83)	13.67 (CI: 2.07, 24.55)	0.23	0.11	<0.001
Speech rate SD (*n* = 15 interactions)	114.87 (CI: 104.13, 121.22)	120.66 (CI: 115.89, 124.21)	5.29 (CI: 0.90, 7.43)	0.31	0.05	<0.001

We observed non-significant trends for the interaction between the two factors (intervention and MS) in speech rate variability, facial expressivity, gesture, and fidgeting.

After the intervention, MS participants (compared to non-MS) displayed higher gesturing (mean difference: 0.58, CI: 0.21 0.95, *R*^2^ marginal = 0.15, *p* = 0.0003) and facial expressivity (mean difference: 0.28, CI: 0.17 0.53, *R*^2^ marginal = 0.01, *p* = 0.029), while non-MS displayed lower fidgeting (mean difference: −0.35, CI: −0.76 0.05, *R*^2^ marginal = 0.06, *p* = 0.05), and higher speech rate variability (mean difference: 6.33, CI: 3.81 8.84, *R*^2^ marginal = 0.09, *p* < 0.0001).

### Impact of intervention on the structure of interactional behavior

We observed a significant correlation between the interlocutors’ facial expressivity, and a marginally significant correlation for gesture. The correlations for fidgeting and head movements were not significant (cf. Table 5). These correlations did not change statistically due to intervention (*p* > 0.28).

**Table 5:** **Mixed effects correlations between interlocutors’ behaviors**.

Feature	*R*	*R*^2^	Marginal *R*^2^	*P*
Gesture (*n* = 30 interactions)	0.16 (CI: −0.04, 0.36)	0.65	0.03	0.071
Fidget (*n* = 30 interactions)	0.06 (CI: −0.21, 0.33)	0.55	0.003	0.57
Facial expressivity (*n* = 30 interactions)	0.27 (CI: 0.08, 0.46)	0.84	0.07	0.0088
Head movement (*n* = 30 interactions)	0.14 (CI: −0.06, 0.32)	0.87	0.001	0.78

We observed a statistical decrease in linguistic coordination after the intervention (cf. Table 6)^2^.

**Table 6:** **Impact of intervention on linguistic coordination**.

Feature	Before intervention	After intervention	Difference	*R*^2^	Marginal *R*^2^	*P*
Pitch RR (*n* = 15 interactions)	0.05 (CI: 0.04, 0.06)	0.04 (CI: 0.03, 0.05)	−0.01 (CI: −0.03, −0.001)	0.37	0.08	0.008
Pitch DET (*n* = 15 interactions)	0.9 (CI: 0.89, 0.92)	0.87 (CI: 0.85, 0.90)	−0.03 (CI: −0.06, −0.01)	0.23	0.05	0.067
Pitch L (*n* = 15 interactions)	5.51 (CI: 4.84, 6.29)	5.02 (CI: 4.33, 5.87)	−0.49 (CI: −1.51, −056)	0.05	0.003	0.47
Pitch LMAX (*n* = 15 interactions)	190.97 (CI: 95.38, 356.78)	164.10 (CI: 101.08, 259.93)	−26.87 (CI: −90.23, 5.72)	0.0001	0.0001	0.90
Pitch ENTR (*n* = 15 interactions)	2.31 (CI: 2.06, 2.56)	2.14 (CI: 19, 2.37)	−0.17 (CI: −0.35, −0.01)	0.003	0.003	0.52
Speech rate RR (*n* = 15 interactions)	0.17 (CI: 0.11, 0.23)	0.05 (CI: 0, 0.11)	−0.06 (CI: −0.12, −0.001)	0.19	0.04	0.009
Speech rate DET (*n* = 15 interactions)	0.77 (CI: 0.60, 0.95)	0.38 (CI: 0.22, 0.54)	−0.29 (CI: −0.45, −0.14)	0.29	0.25	<0.001
Speech rate L (*n* = 15 interactions)	10.94 (CI: 5.76, 16.12)	2.65 (CI: −2.19, 7.49)	−4.15 (CI: −7.42, −0.88)	0.12	0.12	0.009
Speech rate LMAX (*n* = 15 interactions)	71.57 (CI: 48.85, 100.29)	6.50 (CI: −20.37, 33.37)	−21.78 (CI: −47.72, −4.16)	0.06	0.06	0.04
Speech rate ENTR (*n* = 15 interactions)	2.28 (CI: 1.83, 2.72)	0.99 (CI: 0.58, 1.4)	−0.88 (CI: −1.31, −0.44)	0.26	0.26	<0.001

Möbius syndrome participants’ responses to the questionnaires probing social competence and social anxiety revealed no significant differences across the three points (immediately before the experiment, immediately afterward, and 6 months later).

### Relation between interactional behaviors and rapport

Based on interactional behaviors, we can statistically predict coder-assessed rapport. Amount (RR) and stability (L and LMAX) of speech rate coordination were selected by ElasticNet as the minimal set of significant, combined predictors and explained 28% of the variance of rapport: Adj *R*^2^ = 0.28, *p* = 0.0007. In other words, the lesser the coordinated and stable speech rate dynamics, the higher the coder-assessed rapport. Self-reported rapport could not be statistically predicted.

## Discussion

### Summary of the results

Our findings suggest that the intervention had an impact on individual expressive behavior and behavioral alignment. Observer-coded rapport was statistically higher after the intervention, though not self-reported rapport. The increase in observed rapport provides initial support for the prediction that the deliberate employment of compensatory expressive strategies by people with MS can enhance the rapport that they – and their interaction partners – experience in social interactions.

Both interlocutors displayed higher non-verbal expressivity and a more varied speech rate after the intervention. MS participants in particular showed a more marked increase in gesturing and facial expressivity, while non-MS participants showed a more marked decrease in fidgeting and their speech rate became more varied. Matching of non-verbal behavior between interlocutors did not statistically change, while linguistic alignment – statistically present throughout the whole testing – decreased after intervention. Most importantly, the change in alignment seems to be related to the change in experience of the interaction. Externally rated rapport (but not self-reported rapport) was shown to be most strongly – and negatively – correlated with speech rate alignment.

Although these findings generally corroborate the predictions we had articulated, there are several points that merit careful consideration: the lack of results for self-reported rapport, the decrease in linguistic alignment associated with increased rapport, and the limited sample size of the study. We shall now briefly discuss each of these as well as several possibilities for further research that are suggested by our findings.

### The effect of social norms on self-reported rapport

It is interesting to note that *self-reported rapport* did not statistically increase with intervention, and could not be correlated with interactional behaviors, while externally coded rapport could. Personal experience is a crucial dimension to investigate in interactions, and self-report often provides useful information [e.g., Ref. (64)]. However, self-report is often influenced by social norms and by various forms of self-deception (65). One possibility is that both interlocutors may have rated the interaction positively because of politeness norms. Indeed, self-reported rapport was relatively high regardless of whether the intervention had occurred. Non-verbal behavior is considered less susceptible to social desirability and more indicative of internal states because it is more difficult to consciously control than verbal reports (66, 67). So, although more research is needed on getting reliable self-report of rapport, the finding that observer-rated rapport and expressivity improved, and that we could relate changes in alignment to higher observer-rated rapport, suggests we accomplished the more difficult task of improving implicit intergroup rapport.

### Higher rapport is related to decreased interactive alignment

We observed a significant decrease in both pitch and speech rate alignment, but no significant change in non-verbal alignment, and this difference might be due to differences in the dynamics of different behavioral modalities. However, a more plausible explanation lies in the difference in the methods employed to quantify the behaviors and to assess the alignment. Verbal behavior was extracted as a temporal sequence, and analyzed for shared dynamics between interlocutors. Non-verbal behavior was aggregatively assessed in terms of quantity (e.g., of gesture) and analyzed for analogous amounts between interlocutors. The first procedure is more sensitive, and we endeavor to apply analogous measures to non-verbal behavior in future work.

As to the decrease in linguistic alignment and its positive relation to rapport, the finding resonates with a series of recent studies (20, 21, 38, 46, 47, 68) associating alignment with struggles to repair disfluent interactions. Thus, higher alignment in pre-intervention interactions might be an index of higher levels of social impairment, which would decrease after intervention. In support of this interpretation, we observe that both interlocutors – and non-MS participants in particular – show lower degrees of fidgeting and more variance in speech rate after intervention. This could be indicative of a decrease in awkwardness and nervousness. Higher alignment (in the form of more similar temporal dynamics) before intervention might be due partly to awkward and repetitive behaviors.

In sum, our findings add to the growing literature contributing to a richer articulation of current models of alignment: alignment seems to be built upon multiple mechanisms and not to relate in a straightforward fashion to rapport and fluent interactions. It follows that instead of being conceptualized as a simple and automatic mechanism, alignment should be carefully situated in the context of the interaction and its goals and related to the characteristics of the individual behaviors of the interlocutors (20, 38).

### Limitations

The current findings should be considered as pointers for further studies. The small number of available participants with MS in the target age group in Denmark made it very difficult to increase the sample size and to include a control group. We employed conservative statistics accounting for repeated measures and individual variability to better assess effect size uncertainty, and when assessing the relation between behavior and rapport we employed cross-validation to maximize generalizability of the results. However, additional and possibly larger studies are necessary to properly assess the findings, as effect sizes and consequently power analyses are known to be unreliable with small samples (69). Thus, our study provides initial results on which to build future studies, but will have to be re-evaluated in the light of their findings, e.g., via a meta-analysis. Additionally, it is not possible to rule out the possibility that some of our results may be due to a confound, namely that the participants with MS become more confident and/or comfortable because they expected to benefit from the social skills workshop (i.e., rather than because of what they specifically learned and practiced in the workshop), or because they became more comfortable with the procedures as the experiment progressed. We used a different group of non-MS participants on day 2 in order to rule out the possibility that the interactions would run more smoothly simply because non-MS participants became familiar with the procedure. However, as previously argued, individual behaviors resonate in the interaction, making it difficult to clearly separate MS from non-MS participants’ behaviors. A follow-up study, including a control group, would be highly desirable.

### Implications and directions for future research

Practically speaking, the findings that the intervention may have increased compensatory expressivity and behavioral rapport suggest that this program could be valuable in improving social communication for people with MS. Given that the observed behavioral changes were not accompanied by changes in self-reported social competence or social anxiety, it is possible that participants were not conscious of these changes (65). With long-term intervention and with follow-up, perhaps participants’ self-perception of their social skills would improve.

More generally, the intervention may be useful for people with other conditions resulting in facial paralysis, such as Bell’s palsy, Ramsey–Hunt syndrome, stroke, and acoustic neuroma. Indeed, because people who acquired facial paralysis after birth have been found to use less compensatory expression than people with MS (30), these individuals may especially benefit from the intervention. Craniofacial conditions, such as cleft lip and palate, hemifacial microsomia, and facial burns, have long been noted to result in social difficulty. The social challenges associated with these conditions have been generally thought to derive from interlocutors’ responses to an esthetically different face. However, these conditions can distort facial features, skin, or muscles and impair the ability to produce recognizable facial expressions, which may exacerbate social interaction problems. Many other conditions result in reduced expressivity, including depression, schizophrenia, autism, and Parkinson’s disease. Although social functioning difficulty has long been noted in these conditions, there is a paucity of interventions focused on improving expression. As suggested by the current research, increased expression could improve rapport, leading to increased social comfort and belonging.

The decrease in fidgeting and increase in speech rate variability on the part of non-MS participants in our study provides support for the conjecture that some of the difficulties experienced by people with MS in social interactions may arise from other people’s discomfort or uncertainty about how to behave. In other words, people without MS who interact with people with MS may interrupt the smooth flow of interaction through their uncertainty about how to interact in what is for them a new and sensitive situation. Along these lines, social impairment might not be due simply to an impairment, but in important ways also to the context through which the disability is perceived and reacted upon in interactions.

Finally, it would be highly valuable for further research to explore the question as to whether some compensatory strategies (e.g., use of hand gestures, eye contact, and prosody) are more easily automated than others. If there are such differences among the degrees to which different strategies can be automated, this could be important for three concrete reasons. First of all, it may be taxing and distracting to employ deliberate strategies for expressing oneself in social interactions, and people may, therefore, find it tiring, and be less likely to continue doing it [cf. Ref. (19)]. Second, it may be important for some expressive, interactive processes that they occur without people’s awareness. Attempting to bring them about deliberately may actually interfere with the automatic processes that generally bring them about, and could even be counterproductive if it appears forced or unnatural. Third, it would be important for future social skills workshops to examine whether some compensatory strategies are more effectively taught indirectly, or whether some easily automated processes may trigger others. In other words, rather than telling participants to use more gestures or prosody, it may be possible to lead them to do so by some other means which does not require them to deliberately attend to their gestures or prosody, for example, by using more gestures and prosody when interacting with children with MS, by asking them to watch videos in which actors are highly expressive in their gestures and prosody, or by engaging them in role-playing games in which a high level of gesture and/or prosody is appropriate.

## Conclusion

The findings reported here provide evidence that a social skills workshop for teenagers with MS can help to increase the level of rapport they achieve in social interactions. They also provide insight into the mechanisms underlying the increase in rapport. Specifically, the workshop appears not only to have increased the level of expressivity of the participants with MS but also to have led indirectly to an increase in expressivity, and to a decrease in fidgeting and other nervous behavior, in their interaction partners without MS. These results support the idea that one important way of improving social interactions for people with MS (and perhaps for individuals with facial impairment owing to other causes, or with other forms of disability) is to help them to find ways to communicate clearly and put their interaction partners at ease. In addition, the observed decrease in alignment after the intervention, and the negative relation between rapport and alignment provides constraints for theorizing about the mechanisms and functions of alignment processes in social interactions.

## Conflict of Interest Statement

The authors declare that the research was conducted in the absence of any commercial or financial relationships that could be construed as a potential conflict of interest.

## Footnotes

^1^For a similar take on language development in infants with autism spectrum disorder, cf. Warlaumont et al. (18).

^2^These results are not simply due to a change in individual behaviors (e.g., increase in individual variability). To rule these out, we tested surrogate pairs (matching interlocutors from different conversations) before and after intervention and used their level of coordination as baseline for the real pairs. Even in this, more conservative analysis alignment decreased after intervention.
